# Pediatric non-infectious inflammatory lytic lesions of the skull: The pitfalls of diagnosis

**DOI:** 10.1016/j.bonr.2025.101888

**Published:** 2025-11-09

**Authors:** Peggy Alkefrawi, Sylvain Breton, Geneviève Baujat, Isabelle Melki, Benjamin Fournier, Brigitte Bader-Meunier

**Affiliations:** a Department of Pediatrics, Hôpital Saint Camille, Bry sur Marne, 94360, France; b Reference Centre for Rare Systemic Rheumatological and Autoimmune Diseases in Children (RAISE), Hôpital Necker (APHP), Inserm UMR 1163, Paris, 75015, France; c Department of genetics, Imagine Institute, Reference center for constitutional bone diseases, Hôpital Necker (APHP), Paris, 75015, France; d Department of Pediatrics, Hôpital Armand Trousseau (AP-HP), Paris, 75012, France; e Laboratory of Neurogenetics and NeuroinflammationIMAGINE Institut, France; f Department of Paediatric Immunology and Rheumatology, Immunogenetics of Pediatric Autoimmune Diseases, IMAGINE Institute, Reference Centre for Rare Systemic Rheumatological and Autoimmune Diseases in Children (RAISE), Hôpital Necker (APHP), Paris, 75015, France

**Keywords:** Osteitis, Inflammatory lesion, Neurocranium

## Abstract

Chronic non-bacterial osteomyelitis of the skull is a rare entity that should be taken into account in the differential diagnosis of lacunar lesions of the skull. Here, we present four challenging cases of non-infectious inflammatory lytic lesions of the neurocranium with a diagnostic delay of one to five years.

## Highlights

• Chronic non-bacterial osteomyelitis of the skull is a rare entity.
• Brain Imaging and bone biopsy are essential for the diagnosis.
• Searching for extra-cranial involvement by whole body MRI is helpful.

## 1 Introduction

**Fig. 1:**
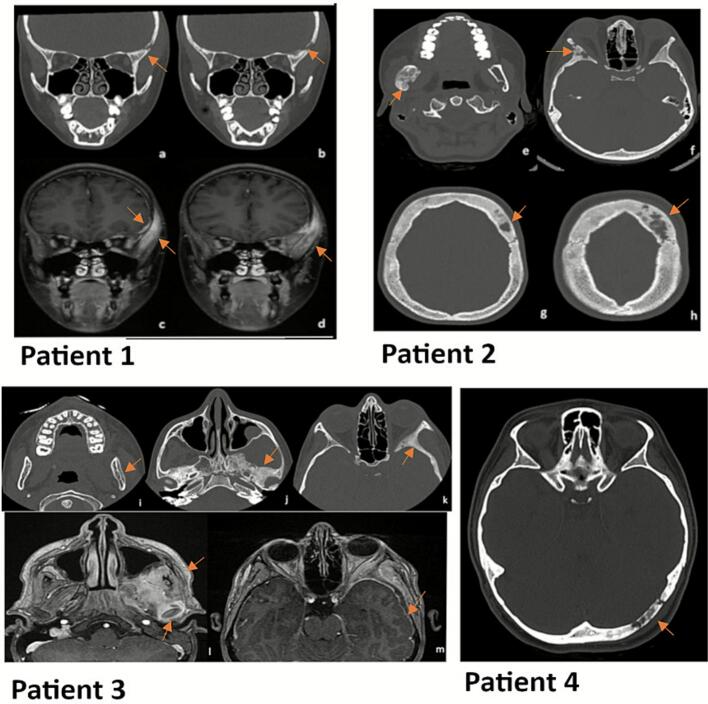
Patient 1: CT scan facial bones, coronal views, bone window: lytic left temporal bone lesion, cortical destruction (a,b). MRI of the face, coronal T1 weighted image with fat signal suppression and Gadolinium enhancement: left temporal bone lesion, adjacent lepto-meningeal thickening, muscular edema and temporo-mandibular synovitis (c,d) Patient 2: CT scan of facial bone and cranium, axial views, bone window: Lesion of the right ascending branch of the mandible, focal osteolysis, cortical destruction, osteosclerosis, unilamellar periosteal reaction (e). Multifocal bone lesions of the right orbit (f) and left frontal bone (g,h), focal osteolysis, cortical destruction and osteosclerosis. Patient 3: CT scan, axial cuts, bone window: Bone lesion of the left ascending mandible with unilamellar periosteal reaction (i). Multifocal lesions of the left sphenoidal bone (j) and orbit (k) with osteolysis, cortical erosion, osteosclerosis, and periosteal reaction. MRI: Lesion with leptomeningeal enhancement and temporo-mandibular synovitis (l, m). Patient 4: CT scan of the facial bones, axial views, bone window: lytic left occipital bone lesion with cortical erosion and adjacent osteosclerosis.

Lytic lesions of the skull are relatively common imaging findings and represent a diagnostic challenge in the pediatric population. They may be normal variants of cranial anatomy or typically result from congenital, traumatic, infectious, vascular disorders or tumors (Santos et al., 2024). Early diagnosis is essential due to the proximity to the soft tissues of the neurocranium, the potential neurological complications and severe potentially urgent diagnoses. We report here the cases of four children with non-infectious inflammatory lytic cranial lesions who were misdiagnosed for several years. These data highlight the importance of a detailed clinical, biological and radiological assessment in children with unexplained lytic lesions of the skull, to avoid unnecessary investigations and misdiagnosis. A bone biopsy is essential to identify the cause of these lesions in atypical presentations or remaining doubts. (See Fig. 1.) (See Table 1.)

**Table 1:** Demographic, clinical, immunological and radiological characteristics of the 4 patients at diagnosis, treatment and follow up.

	Patient 1	Patient 2	Patient 3	Patient 4
Medical history	None	None	None	None
Age at first manifestation (years)/Sex	11/F	10/F	13/M	10/F
Revealing manifestations	Left temporal headache	Bilateral palpebral swelling Frontal headache	Left palpebral swelling Lowering of the eyeball	Left-sided frontal headache
Initial diagnosis	Skull malignant tumor	Bone fibrous dysplasia	Skull malignant tumor	Langerhans cell histiocytosis
Time elapsed from the initial diagnosis to the diagnosis of inflammatory osteitis	1 year	5 years	1 year	1 year
CRP values (mg/L) at onset of the disease (*N* < 5 mg/L)	8.5	88	4	6.4
Cerebral MRI findings	Lytic left temporal bone lesion with adjacent lepto-meningeal thickening and muscular edema	Lytic bone lesions of the right orbit, right ascending branch of the mandible and left frontal bone	Lytic lesions of the left sphenoid bone and the left zygomatic bone. Thickening of the left ascending branch of the mandibular bone Left temporo-mandibular synovitis	Lytic lesion of the left occipital bone
Whole body MRI	Not done	No additional pathological findings outside the skull.	Edema of the distal metaphyses of the right femur	Left occipital lytic lesion Sacral, ischial, right femoral trochanteric, sternal, bilateral wrists, right knee and right foot lesions
Biopsy findings	Neo-osteogenesis of periosteal origin associated with fibrosis and lympho-plasmocytic infiltrate: aspect of chronic osteitis. Sterile culture. Negative markers (CD1a, CD5, CD10, CD20, CD30, CD79a, desmin, myogeninm synaptophysin, TdT, CD99, ki76, anti MDM2, HMGA2)	First biopsy: Numerous myo-fibroblasts, negative markers (CD1a, CD34, CD99 and protein S) Second biopsy 5 years later: Chronic osteitis with plasmocytic predominance. Sterile culture	Lympho-Plasmatic infiltrates associated with fibrinous material. Sterile culture. Negative pathologic markers.	Abundant polymorphic inflammatory infiltrate with neutrophil predominance, associated with histiocytes, lymphocytes and plasmocytes. Osteolytic lesions with inflammatory acute and chronic osteitis aspect. Absence of necrosis. Absence of bacterial pathogen. Rare CD1a + cells.
Treatment	NSAIDS	NSAIDS and Etanercept Switched to adalimumab due to inefficient efficacity	Etanercept	NSAIDS
Clinical remission at last follow-up (Time elapsed from onset of treatment to last follow-up (months))	Yes (25)	Persistence of bone pain on NSAIDS and Etanercept. Remission on Adalimumab (5)	Yes (29)	Yes (24)

## 2 Cases

We retrospectively reviewed the charts of all patients with lytic skull lesions who were followed up between December 2010 and December 2024 at the Paris Referral Center for Rare Pediatric Rheumatism and Systemic Autoimmune Diseases (RAISE). After parental consent, patients were registered in the French rare disease data center (BAMARA).This retrospective observational study complied with ethical guidelines and institutional regulation involving human participants (authorization to collect data CNIL n°1,980,120).

Four patients aged 10 to 13 years at first manifestation were included. Demographic, clinical, laboratory findings, histology and radiological characteristics at diagnosis are shown in the Table.

The presenting symptom was headache in all but one patient. The time from the first manifestation to the diagnosis of osteitis ranged from 1 to 5 years. The radiological manifestations are described in the Table and Figure.

Patient 1 presented with an isolated lytic lesion of the temporal bone (Figure).

Patient 2 was initially misdiagnosed with fibrous dysplasia (FD) for 5 years, and the skull lesions were associated with lytic lesions of the mandible, orbital, and frontal bones on cranial MRI (Figure). The diagnosis of FD was reconsidered because of persistent severe headaches and elevated C-reactive protein (CRP) that was not explained by a bacterial infection or any other inflammatory condition.

Patient 3 had lytic lesions involving the sphenoid and zygomatic bones, along with thickening of the ascending branch of the mandible and temporomandibular joint synovitis and arthritis on cranial MRI (figure).

Patient 4, who initially presented with left sided frontal headache, had a lytic lesion of the left occipital bone (figure), with whole-body MRI showing multiple metaphyseal bone lesions in the sacral, ischial, right femoral trochanter, sternum, bilateral wrists, right knee, and right foot regions.

CRP levels were slightly increased in patient 1 and 4, markedly increased in patient 2, and normal in patient 3.

In all patients, a non-infectious osteitis was diagnosed by bone biopsy, which showed a lympho-plasmocytic infiltrate without markers suggestive of Langerhans cells or infection. The four patients were followed-from 3 to more than 24 months.

All the patients were symptoms-free at the last follow-up either on non-steroidal anti-inflammatory drugs (NSAIDs) (patients 1 and 4), or on anti-TNF treatment (etanercept in Patient 3 and adalimumab in Patient 2) (Table). Bone lesions improved in all the patients on brain MRI or on a full body MRI from 8 to 18 months after the initiation of treatment.

## 3 Discussion

These cases highlighted the clinical challenges of diagnosing chronic non-bacterial osteomyelitis (CNO) of the skull. Sterile bone inflammation covers a large clinical spectrum with self-limited mono -focal inflammatory bone involvement at one end of the spectrum and chronic recurrent multi-focal bone lesions at the other end, which are then referred to as chronic recurrent multifocal osteomyelitis in the pediatric population (CRMO) (Fraleigh et al., 2023). For our patients, we use the term CNO, since not all the patients presented with multifocal lesions. These diseases are characterized by a non-specific infiltrate of neutrophils and plasmocytes on biopsy. CNO is a rare autoinflammatory bone disease with typical onset in childhood. It may manifest only as bone lesions, but may be associated with other inflammatory conditions, such as psoriasis or Sweet's syndrome and inflammatory bowel disease (Fraleigh et al., 2023; Zhao et al., 2021).

As observed in our patients, the most common symptom of CNO is bone pain, sometimes associated with swelling. Laboratory findings are nonspecific, as inflammatory markers may be normal. Targeted plain radiographs are performed to rule out other diagnoses, especially malignant bone tumor: they may show lytic bone lesions, sclerosis and hyperostosis, but may also be normal at the early stages of CNO. Magnetic resonance imaging is a key tool in the differential diagnosis and for the screening of clinically silent lesions. It may show metaphyseal bone and soft tissue inflammation, associated bone destruction, periosteal reaction, hyperostosis, growth plate damage and vertebral fracture. Soft tissue involvement surrounding the affected bones has also been described. The characteristic bone lesions of CNO are most commonly found in the metaphysis of the long bones of the lower and upper extremities, followed by the vertebrae and clavicles, and typically do not involve the skull (Fraleigh et al., 2023). In fact, to the best of our knowledge, there are only ten previously reported cases of CNO involving the skull (Koizumi et al., 2012; Wedman and van Weissenbruch, 2005; Watanabe et al., 2015; Barrani et al., 2015; Sia et al., 2017; Bustamante et al., 2021; Mudri et al., 2022).

The pathophysiology of CNO is poorly understood, but it has been shown to involve an imbalance in cytokine production with increased pro-inflammatory cytokines and lack of expression of cytokines that modulate the immune response, leading to bone inflammation. However, CNO remains a diagnosis of exclusion. There are No widely accepted and prospectively tested diagnostic criteria available (Zhao et al., 2021). Bone biopsy is often performed to rule out bacterial osteomyelitis, Langerhans cell histiocytosis, and neoplasia, especially in the case of a unique or atypical bone lesion. In the early stages of CNO, neutrophilic and lymphocytic infiltrates are seen. Our patients responded to nonsteroidal anti-inflammatory drugs and anti-TNF agents, which are commonly used as first- and second-line treatments respectively (Zhao et al., 2021). However spontaneous remission cannot be ruled out.

The differential diagnosis of lytic bone lesions of the skull in pediatrics is broad and typically includes infections, benign tumors (Langerhans cell histiocytosis, fibrous dysplasia), malignant neoplastic lesions (neuroblastoma, especially neuroblastoma metastases), hematological malignancies, scurvy, some genetic diseases (autoinflammatory diseases with bone involvement, hypophosphatasia and doughnuts lesions), post-traumatic, and congenital lesions (Santos et al., 2024; Zhao et al., 2021). Here we emphasize that inflammatory osteitis should be added to the differential diagnosis of lytic lesions of the skull (Fraleigh et al., 2023). Careful physical examination, whole blood cell count, CRP levels, and cranial imaging are key elements in the diagnostic process. Early whole-body MRI to identify potentially asymptomatic bone lesions and careful examination of the temporomandibular joint should be considered in the diagnosis of CNO in a child with a lytic skull lesion.

Non-infectious inflammatory lesions of the skull should be included in the differential diagnosis of lytic lesions of the skull. Careful search for extracranial bone involvement by whole-body MRI may be helpful in differentiating cranial osteitis from its mimics.

## CRediT authorship contribution statement

**Peggy Alkefrawi:** Writing – review & editing, Writing – original draft, Supervision, Project administration, Methodology, Data curation, Conceptualization. **Sylvain Breton:** Writing – review & editing, Methodology, Formal analysis, Data curation. **Geneviève Baujat:** Writing – review & editing, Methodology, Formal analysis, Data curation. **Isabelle Melki:** Writing – review & editing, Methodology, Formal analysis, Data curation. **Benjamin Fournier:** Writing – review & editing, Methodology, Formal analysis, Data curation. **Brigitte Bader-Meunier:** Writing – review & editing, Supervision, Project administration, Methodology, Data curation, Conceptualization.

## Consent

Written informed consent for publication of this case report was obtained from the patients' legal guardians.

## Funding

This research did not receive any specific grant from funding agencies in the public, commercial, or not-for-profit sectors.

## Declaration of competing interest

The authors have no conflicts of interest to disclose.

## Data Availability

Data will be made available on request.
